# Sustained favorable long-term outcome in the treatment of schizophrenia: a 3-year prospective observational study

**DOI:** 10.1186/1471-244X-11-143

**Published:** 2011-08-26

**Authors:** Gebra B Cuyún Carter, Denái R Milton, Haya Ascher-Svanum, Douglas E Faries

**Affiliations:** 1 All authors are employees of Eli Lilly and Company, Global Health Outcomes; Indianapolis, IN, USA 46285

## Abstract

**Background:**

This study of chronically ill patients with schizophrenia aimed to identify patients who achieve sustained favorable long-term outcome - when the outcome incorporates severity of symptoms, level of functioning, and use of acute care services - and to identify the best baseline predictors of achieving this sustained favorable long-term outcome.

**Methods:**

Using data from the United States Schizophrenia Care and Assessment Program (US-SCAP) (N = 2327), a large 3-year prospective, multisite, observational study of individuals treated for schizophrenia in the US, a hierarchical cluster analysis was performed to group patients based upon baseline symptom severity. Symptom severity was assessed using the Positive and Negative Syndrome Scale (PANSS) scores, level of functioning, and use of acute care services. Level of functioning reflected patient-reported productivity and clinician-rated occupational role functioning. Use of acute care services reflected self-reported psychiatric hospitalization and emergency service use. Change of health state was determined over the 3-year period. A patient was classified as having a sustained favorable long-term outcome if their health state values had the closest distance to the defined "best baseline cluster" at each point over the length of the study. Stepwise logistic regression was used to determine baseline predictors of sustained favorable long-term outcome.

**Results:**

At baseline, 5 distinct health state clusters were identified, ranging from "best" to "worst." Of 1635 patients with sufficient data, only 157 (10%) experienced sustained favorable long-term outcome during the 2-years postbaseline. The baseline predictors associated with sustained favorable long-term outcome included better quality of life, more daily activities, patient-reported clearer thinking from medication, better global functioning, being employed, not being a victim of a crime, not having received individual therapy, and not having received help with shopping and leisure activities.

**Conclusions:**

Only a small percentage of patients achieved sustained favorable long-term outcome in this study, suggesting there continues to be a great need for improvement in the treatment of schizophrenia. Findings suggest that clinicians could make early projections of health states and identify those patients more likely to achieve favorable long-term outcomes enabling early therapeutic interventions to enhance benefits for patients.

## Background

Heterogeneity of response and outcome is common among patients treated for schizophrenia [1]. Clinical study results indicate that about 70% of patients fail to experience at least minimal efficacy early in treatment [2,3], and current medications are effective for approximately 50% of patients [4-6]. Poor efficacy can lead to early treatment discontinuation, exacerbation of symptoms, relapse, and increased hospitalization with higher treatment costs [7-10].

A recent study exploring treatment response trajectories in schizophrenia using data from clinical trials found that 77% of patients were classified as moderate responders, 8% as poor responders, and 15% as rapid responders [11]. A study that used hospitalization as a proxy measure for psychotic symptom exacerbation over a 10-year period found schizophrenia amelioration in approximately 75% of patients, deterioration in approximately 25% of patients, and stability in less than 1% of patients [12]. These results underscore the need to better understand patients' heterogeneity to help improve patient long-term outcomes.

It has been suggested that the definition of "outcome" in schizophrenia may need to be broadened beyond symptom severity to also include quality of life, subjective well-being, health status, use of healthcare services, and measures of the patients' level of functioning [13-15]. Capturing multiple domains is important to assess the patient holistically and at varying stages of the illness. When outcome is broadly defined - beyond symptom improvement - relatively little is known about the baseline characteristics that can be used to predict a favorable long-term outcome among chronically ill patients with schizophrenia who are treated in usual care settings.

Using data from a large 3-year observational naturalistic noninterventional study in the United States, this analysis aimed to identify distinct health states among chronically ill patients with schizophrenia, using a broad definition of health state that incorporated severity of symptoms, level of functioning, and use of acute care services. Employing these health states, which varied from "best" to "worst," the second part of the analysis aimed to identify patients who achieved sustained favorable long-term outcome and the best baseline predictors of this favorable health state.

## Methods

### Data Source

The data source for this study was the United States (US) Schizophrenia Care and Assessment Program (SCAP), a 3-year prospective, observational study (N = 2327). Participants were adults 18 years and older and treated for schizophrenia, schizoaffective, or schizophreniform disorders, based on Diagnostic and Statistical Manual of Mental Disorders, fourth edition (DSM-IV) criteria. The study was conducted between July 1997 and September 2003, and the sample was geographically and ethnically diverse, representing treatment in large systems of care. Patients were recruited from community mental health centers, university healthcare systems, community and state hospitals, and the Department of Veterans Affairs Health Services [16]. The overall objective of US-SCAP was to better understand the treatment of patients with schizophrenia in usual care settings. Patients were excluded if they were unable to provide informed consent or had participated in a clinical drug trial within 30 days prior to enrollment. Enrollment was not contingent upon being treated with a specific antipsychotic or with any medication. Patients could continue with medications they received prior to enrollment for as long as necessary, and decisions about medication changes, if any, reflected those made by physicians and their patients, as they naturally occur in usual practice. Almost all study participants were outpatients at the time of enrollment (93.5%). Of 2327 participants, most completed 1 year of follow-up (78.1%), with fewer completing 2 years (69.6%) and 3 years (65.2%). At enrollment, almost all patients (94.7%) were treated with at least one antipsychotic medication, including oral typical (36.7%), oral atypical (58.1%), and depot typical antipsychotics (19.6%). Treatment throughout the study was based on physicians' decisions, which could include medication augmentation, switching, or discontinuation, reflecting the dynamic antipsychotic treatment observed in naturalistic care settings. Institutional Review Board (IRB) approval was obtained at each regional site prior to initiation of the study, and the study was conducted in accordance with the ethical principles that have their origin in the Declaration of Helsinki and are consistent with good clinical practices and applicable laws and regulations. Informed consent was received from all participants.

### Measures

This study used a number of clinician-rated and patient-reported measures in addition to patients' medical records. Patients' medical records provided information about healthcare utilization, such as psychiatric hospitalizations and medications (i.e., antipsychotics, antidepressants, mood stabilizers, antiparkinsonian agents, and mood stabilizers). This information was systematically collected using the Medical Records Abstraction Form (MRAF). Information about functional and quality-of-life outcomes was derived from the SCAP Health Questionnaire (SCAP-HQ) [17]. This 102-item structured interview was developed for the US-SCAP study and was administered to patients at enrollment and at 6-month intervals thereafter. Items for the SCAP-HQ were drawn from existing measures, such as the Lehman Quality of Life Interview [18], the Arkansas Schizophrenia Outcomes Module [19,20], the Medical Outcome Study Short Form-12 (SF-12) [21], and the CAGE, a screening tool for assessment of alcohol-related problems [22]. The psychometric properties of the SCAP-HQ were found to be acceptable for application to large-scale studies in routine care based on a study of its internal consistency, convergent validity, test-retest reliability, and responsiveness to change.

Patient symptoms of schizophrenia and depressive symptoms were assessed annually by a clinician using the Positive and Negative Syndrome Scale (PANSS) [23] and the Montgomery-Åsberg Depression Rating Scale (MADRS) [24], respectively. Clinicians also annually assessed medication-emergent adverse events, including extrapyramidal side effects using the Simpson-Angus Scale [25] and tardive dyskinesia using the Abnormal Involuntary Movement Scale (AIMS) [26]. In addition to using the SCAP-HQ to evaluate both patient-reported level of functioning and quality of life, clinicians also used the Global Assessment of Functioning (GAF) [27] to evaluate level of functioning and the Quality of Life Scales (QLS) [28] to evaluate quality of life.

Socio-demographic information data were collected at enrollment and included age, gender, race, marital status, education, employment, and insurance status. In addition, DSM-IV diagnosis of schizophrenia (i.e., schizophrenia, schizoaffective, or schizophreniform) and age of illness onset were included. The remaining measures investigated in this analysis are described in Table 1.

**Table 1:** Description of Measures

MEASURE	SOURCE	DESCRIPTION
**SOCIO-DEMOGRAPHICS**		

Family history	Screening interview	History of emotional or psychiatric illness for any of the following family members: parent, sibling, child, grandparent, aunt, uncle, cousin, or distant relative
Supervised housing	SCAP-HQ	Includes in house/apartment where mental health professionals visit, in program with mental health professionals there most of the time, in a hospital or nursing home, or in jail or prison

**DISEASE-RELATED AND SYMPTOMS**		

Depression	SCAP-HQ	Bothered much by feeling low in energy or slowed down, feeling unhappy, sad, or blue, feeling hopeless about the future, or feeling like a good or worthless person in the past 4 weeks
MADRS total	MADRS	Combines apparent sadness, reported sadness, inner tension, reduced sleep, reduced appetite, concentration difficulties, lassitude, inability to feel, pessimistic thoughts, and suicidal thoughts
Remission	PANSS	A mild, minimal, or absent response to the lack of spontaneity and flow of conversation, conceptual disorganization, delusions (general), unusual thought content, passive/apathetic social withdrawal, hallucinatory behavior, blunted affect, and stereotyped thinking items of the PANSS
PANSS anxiety/depression (Marder)*	PANSS	Combines the disorientation, difficulty in abstract thinking, lack of judgment and insight, and hostility items of the scale
PANSS disorganized (Marder)*	PANSS	Combines the poor rapport, somatic concern, excitement, tension, mannerisms and posturing, uncooperativeness, and disturbance of volition items of the scale
PANSS hostility (Marder)*	PANSS	Combines the anxiety, suspiciousness, emotional withdrawal, and poor attention items of the scale
PANSS negative (Marder)*	PANSS	Combines the passive/apathetic social withdrawal, active social avoidance, poor impulse control, hallucinatory behavior, depression, blunted affect, and preoccupation items of the scale
PANSS positive (Marder)*	PANSS	Combines the lack of spontaneity and flow of conversation, conceptual disorganization, delusions, unusual thought content, guilt feelings, grandiosity, stereotyped thinking, and motor retardation items of the scale
PANSS Bell factor	PANSS	Combines the conceptual disorganization, difficult in abstract thinking, lack of judgment and insight, stereotyped thinking, and poor attention items of the scale
Psychosis	SCAP-HQ	Bothered much by feeling that others are spying against you or plotting against you, hearing voices that other people do not hear, feeling like someone is controlling your thoughts/movements, feeling that you are watched or talked about by others, or feeling like other people are aware of your private thoughts in the past 4 weeks
Vitality	SCAP-HQ	Bothered much by feeling low in energy or slowed down in the past 4 weeks

**FUNCTIONING/BEHAVIORS**		

Arrested	SCAP-HQ	Arrested or picked up for any crime in the past 6 months
Daily Activity	SCAP-HQ	Frequency of taking responsibility for your laundry, doing or helping with household chores, preparing at least simple meals, planning or purchasing food and household items, or shopping for personal necessities in the past 4 weeks
Global assessment of functioning	GAF	Global assessment of patient functioning rating considering psychological, social, and occupational functioning on a hypothetical continuum of mental health illness
Health status	SCAP-HQ	Overall impression of general health (poor, fair, good, very good, or excellent)
Helped by anyone	SCAP-HQ	Received help with household chores, shopping, paying bills, finding a job, getting benefits (i.e., SSI, VA, food stamps, other), talking with lawyers, police, fire, or court officials, or leisure or social activities in the past 4 weeks
Leisure activity	SCAP-HQ	Went shopping, ate at a restaurant or coffee shop, did something fun (e.g., hobby, sports, crafts, etc.), or prepared food for yourself in the past 4 weeks
Mental and physical health (SF-12)	SCAP-HQ	Combines the bodily pain, general health, mental health, physical functioning, role limitations-emotional, role limitations-physical, social functioning, and vitality domains of the SF-12 health survey
Productivity*	SCAP-HQ	Worked at a job for pay, volunteered, attended school, or kept house/took care of children in the past 4 weeks
Social activity	SCAP-HQ	Frequency of doing things with friends, doing something with another person that you planned ahead of time, or spending time with someone more than a friend, boyfriend, girlfriend, or spouse in the past 4 weeks
Social relationships	SCAP-HQ	Frequency of doing things with friends or doing something with another person that you planned ahead of time in the past 4 weeks
Substance abuse	SCAP-HQ	Frequency of having at least a little to drink or using illegal or "street" drugs in the past 4 weeks
Suicide	SCAP-HQ	Thought or talked about hurting or killing yourself or actually attempted to hurt or kill yourself in the past 4 weeks
Victim	SCAP-HQ	Been a victim of a violent crime (e.g., assault, rape, mugging, or robbery) or nonviolent crime (e.g., theft or being cheated) in the past 4 weeks
Violent	SCAP-HQ	Struck or injured someone or threatened to strike or injure someone and meant it in the past 4 weeks
Satisfaction with basic needs	SCAP-HQ	Combines the patient's feeling about the amount of privacy where they live, the way things are in general between them and their family, and the protection they have against being robbed or attacked
Satisfaction with social life	SCAP-HQ	Combines the patient's feeling about the way they spend their time, the amount of fun they have, and the amount of friendships in their life
General life satisfaction	SCAP-HQ	The patient's feeling about their life in general (combining satisfaction with social life and basic needs)
Quality of life scale - item 9*	QLS	Extent of occupational role functioning
Quality of life scale - item 10*	QLS	Level of accomplishment
Quality of life scale total	QLS	Combines intimate relationship with household members, intimate relationships with people other than immediate family or household members, active acquaintances, level of social activity, involved social network, social initiatives, social withdrawal, socio-sexual relations, extent of occupational role functioning, level of accomplishment, degree of underemployment, satisfaction with occupational role functioning, sense of purpose, degree of motivation, curiosity, anhedonia, time utilization, commonplace objects, commonplace activities, capacity for empathy, and capacity for engagement and interaction with interviewer

**HEALTHCARE RESOURCE UTILIZATION**		

Case management	MRAF	Case management (documented in medical record within the past 6 months)
Crisis call	SCAP-HQ	Called a crisis hotline in the past 4 weeks
Emergency service use*	SCAP-HQ MRAF	Had an unscheduled emergency visit with a psychiatrist or therapist in the past 4 weeks Emergency room visit (past 6 months)
Individual therapy	MRAF	Received individual therapy (past 6 months)
Number of hospitalizations/total number of days hospitalized (6 months)	MRAF	Used admission and discharge dates reported on the medical record extraction form
Psychiatric hospitalizations (4 weeks)*	SCAP-HQ	Stayed overnight in a hospital for a mental or emotional problem
Psychiatric hospitalizations (1 year)	Screening interview	Been in the hospital for a mental or emotional problem in the last year

**MEDICATION ADHERENCE**		

Medication possession ratio	MRAF	The cumulative number of days the patient had been prescribed any antipsychotic drug divided by the number of days in the assessment period multiplied by 100
Non-adherence	SCAP-HQ	How regularly did the patient take the medication they were given for mental, emotional, or nervous problems in the past 4 weeks

**MEDICATION-EMERGENT EVENTS**		

Level of abnormal involuntary movements	AIMS	Combines facial and oral movements (muscles of facial expression, lips and perioral area, jaw, tongue), extremity movements (upper [arms, wrists, hands, fingers], lower [legs, knees, ankles, toes]), and trunk movements (neck, shoulders, hips)
Clearer thoughts from medication	SCAP-HQ	Current medication for mental, emotional, or nervous problem is making your thoughts clearer
Medication effects	SCAP-HQ	Current medication for mental, emotional, or nervous problem is making your thoughts clearer, making you feel tired and sluggish, interfering with your normal thinking, making you feel restless, or interfering with your normal sexual functioning
Tardive dyskinesia	AIMS	A response of moderate or severe on either facial and oral movements (muscles of facial expression, lips and perioral area, jaw, tongue), extremity movements (upper [arms, wrists, hands, fingers], lower [legs, knees, ankles, toes]), or trunk movements (neck, shoulders, hips) or a response of mild, moderate, or severe on any 2 of the previous items
Psuedo-parkinsonian symptoms	SA	Combines gait, arm dropping, shoulder shaking, elbow rigidity, fixation of position or wrist rigidity, leg pendulousness, glabella tap, tremor, and salivation
Restlessness	SCAP-HQ	Medication for mental, emotional, or nervous problem is making you feel restless

Abbreviations: AIMS = abnormal involuntary movement scale, BDCF = baseline demographic collection form, GAF = global assessment form, MADRS = Montgomery-Åsberg depression rating scale, MRAF = medical record assessment form, PANSS = positive and negative syndrome scale, QLS = quality of life scale, SA = Simpson-Angus scale, SCAP-HQ = schizophrenia care and assessment program-health questionnaire; SF = short form.

* Measures used in the schizophrenia health state definition.

NOTE: The various patient-reported and clinician reported measures in addition to the items obtained from the medical records investigated in this analysis.

The objectives of this study were: 1) to identify patients with schizophrenia who experience sustained favorable long-term outcome when the outcome incorporates severity of symptoms, level of functioning, and use of acute care services and 2) to identify the baseline measures that predict sustained favorable long-term outcome.

### Definition of Schizophrenia Health State and Sustained Favorable Long-Term Outcome

The first step in this retrospective analysis was to define each patient's health state at baseline using symptom severity, level of functioning, and utilization of acute care services in a cluster analysis. Symptom severity was based on PANSS factor subscale scores [29]: PANSS positive, PANSS negative, PANSS hostility, PANSS disorganized thinking, and PANSS anxiety/depression. The level of functioning reflected patient-reported productivity (SCAP-HQ; composite measure of reported working for pay, volunteering, attending school, and keeping house or taking care of children) and clinician-rated occupational role functioning (QLS item 9) and level of accomplishment (QLS item 10). Acute care services included self-reported psychiatric hospitalization (in the previous 4 weeks) or use of emergency services (emergency room use in the previous 6 months from the medical record or self-reported emergency visit with a psychiatrist in the previous 4 weeks).

Once the health states had been defined by the cluster analysis, the next step included identifying those with sustained favorable long-term outcome, which was the main outcome of interest. A patient was classified as having sustained favorable long-term outcome if they were in the "best" cluster (i.e., experienced the lowest symptom severity and the highest level of functioning) over a 2-year period postbaseline assessment (from year 1 to year 2 and from year 2 to year 3, as assessments were conducted annually postbaseline). Change over time was ascertained by shifts in clusters from baseline to each postbaseline visit (end of year 1, 2, and 3). The last step in the retrospective analysis was to identify baseline measures that were associated with sustained favorable long-term outcome.

### Statistical Methods

As mentioned above, the first step was to define each patient's health state at baseline. This was determined by a hierarchical cluster analysis, using the Ward's minimum variance method [30], of patients' schizophrenia health states to categorize patients into distinct groups at baseline. Postbaseline clusters were defined by first performing a principal component analysis on the 10 health state measures for data at baseline and each postbaseline visit. The "center" for each of the baseline clusters was defined by computing a mean score for each of the resulting 10 principal components at baseline by cluster. Then Euclidean distances were calculated from the "center" of each of the baseline clusters to each patient's 10 principal components at postbaseline. Finally, each patient's postbaseline cluster assignment was determined based on their closest Euclidean distance to each of the clusters at baseline. Patients were required to have nonmissing data for all health state measures (i.e., PANSS subscale scores, QLS items 9 and 10, psychiatric hospitalizations, and emergency services) to be included in the cluster analysis at each time point.

In addition to characterizing patients by sustained favorable long-term outcome in the second step of the analysis, cluster shifts were explored during the three-year period. Improvement of outcome was based on changes to a better cluster from baseline to 1-year postbaseline and maintaining the same improved cluster or moving to an even better cluster the following 2 years. Worsening of outcome was based on changes to a worse cluster from baseline to 1-year postbaseline and staying in that cluster or shifting to an even worse cluster the following 2 years. Patients who did not experience improvement or worsening of outcome were classified as having "no sustained shift in outcome."

Comparisons of baseline characteristics between patients with and without sustained favorable long-term outcome were performed using Fisher's exact tests (categorical) and analysis of variance (continuous). Stepwise logistic regression, following 5 multiple imputations of missing values, was used to determine baseline factors associated with sustained favorable long-term outcome. A total of 62 variables, including the patient-reported variables, clinician-rated variables, and medical record-based resource utilization, were explored. The interdependent variables (variance inflation factor > 10) were removed. A 2-tailed significance level of 0.05 was used to determine whether a baseline measure was included in or excluded from the model.

## Results

Most (83% or 1942/2327) study enrollees had sufficient baseline data for inclusion in the cluster analysis. A baseline comparison of the patients included in the cluster analysis and those not included revealed that the included patients were significantly older (42.2 years versus 40.3 years; p = .0029) and less likely to be unemployed (77.6% versus 83.4%; p = .0122) and had lower PANSS positive scores (17.9 versus 19.5; p < .0001), lower PANSS negative scores (17.8 versus 19.1; p = .0002), lower PANSS hostility scores (10.4 versus 11.3; p < .0001), lower PANSS anxiety/depression scores (10.4 versus 11.2; p < .0001), and higher GAF scores (43.7 versus 33.7; p < .0001).

There were 5 distinct health state clusters identified (Table 2) in the first step of the analysis and labeled from the "best" to "worst" cluster, with severity of symptoms and level of functioning influencing cluster order. The majority of patients (77%) belonged to either the "best" (n = 503) or the "second best" (n = 992) clusters at baseline. Although the average symptom severity and level of functioning was worse for patients in the "worst" cluster, all of the acute care services were experienced by patients in the "middle" and "second worst" groups.

**Table 2:** Baseline Characteristics for Variables Used to Define Health States by Cluster (n = 1942)

Cluster Variables	Best n = 503	Second Best n = 992	Middle n = 145	Second Worst n = 53	Worst n = 249	Total (n = 1942)
**SYMPTOM SEVERITY^a^**						

PANSS positive, mean (sd)	13.81 (3.92)	18.02 (5.38)	19.24 (6.70)	19.72 (6.32)	24.34 (5.27)	17.88 (6.05)
						
PANSS negative, mean (sd)	13.17 (4.06)	17.98 (5.33)	20.08 (6.48)	20.17 (5.99)	24.54 (5.77)	17.79 (6.25)
						
PANSS hostility, mean (sd)	7.77 (2.43)	10.57 (3.35)	11.53 (3.86)	12.08 (3.10)	14.32 (2.79)	10.44 (3.68)
						
PANSS disorganized thinking, mean (sd)	10.66 (2.67)	12.99 (3.64)	13.89 (4.65)	14.43 (3.80)	19.75 (3.97)	13.36 (4.46)
						
PANSS anxiety/depression	8.61 (2.88)	10.51 (3.06)	9.91 (3.16)	10.76 (3.36)	13.61 (3.10)	10.38 (3.37)

**FUNCTIONING**						

Occupational role functioning (QLS 9), mean (sd)	3.59 (1.55)	1.66 (1.62)	1.65 (1.73)	1.34 (1.34)	0.46 (0.80)	1.99 (1.83)
						
Level of accomplishment (QLS 10), mean (sd)	4.39 (1.08)	2.07 (1.54)	2.10 (1.84)	1.58 (1.41)	0.68 (0.91)	2.48 (1.84)
						
Productivity, n (%)	503 (100%)	605 (61)	90 (62.1)	38 (71.7)	92 (36.9)	1328 (68.4)

**ACUTE CARE**						

Emergency use^b^, n (%)	0	0	145 (100)	18 (34)	0	163 (8.4)
						
Psychiatric hospitalizations, (past 4 weeks), n (%)	0	0	0	53 (100)	0	53 (2.7)

Abbreviations: n = number of patients; PANSS = positive and negative syndrome scale; QLS = Quality of Life Scale; sd = standard deviation.

^a ^PANSS factors per Marder et al. (1997) [29]

^b ^Emergency use was both patient reported for the past 4 weeks and from the past 6 months recorded in the medical record.

NOTE: There were 5 distinct outcome clusters identified and labeled from the "best" to "worst" cluster, with severity of symptoms and level of functioning influencing cluster order. The majority of patients (77%) belonged to either the "best" (n = 503) or the "second best" (n = 992) clusters at baseline.

**Figure 1:**
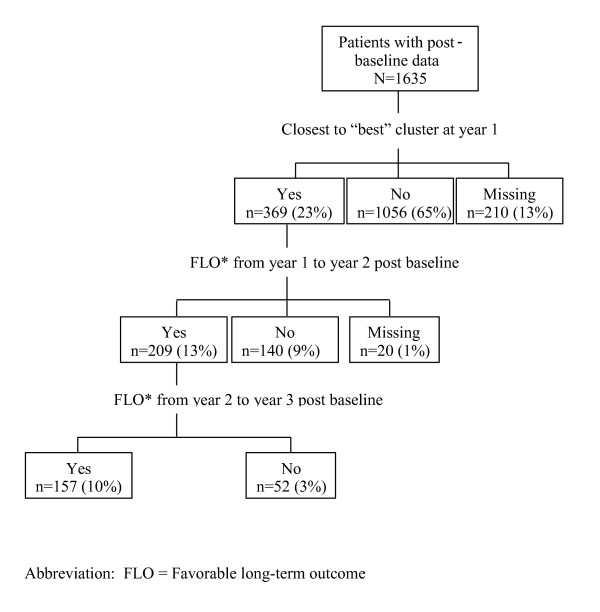
Favorable long-term outcome (FLO*) over time. Of the 1635 patients included in the analysis, 369 (23%) were closest to the "best" cluster at year 1; 209 (13%) achieved favorable outcome over 1 year (from year 1 to year 2); and 157 (10%) achieved favorable sustained outcome over 2 years (from year 1 to year 3).

Approximately 70% of the patients had postbaseline data to examine sustained favorable long-term outcome for the second step of the analysis. A baseline comparison of these patients (n = 1635) and those not included (n = 692) revealed that the included patients were older (42.3 years versus 40.8 years; p = .0039), had higher PANSS positive scores (18.5 versus 17.3; p < .0001), higher PANSS negative scores (18.3 versus 17.3; p = .0007), higher PANSS disorganized scores (13.7 versus 12.8; p < .0001), higher PANSS hostility scores (10.8 versus 10.2; p = .0004), lower GAF scores (41.6 versus 43.3; p = .0063), and lower mean QLS total scores (2.8 versus 3.0; p = .0216). Of the 1635 patients included in the analysis, 369 (23%) were closest to the "best" cluster at year 1; 209 (13%) achieved favorable outcome over 1 year (from year 1 to year 2); and 157 (10%) achieved favorable sustained outcome over 2 years (from year 1 to year 3) (Figure 1).

An assessment of cluster shift over time was conducted to further understand change over the 3-year period, and patients were classified as "improved," "worsened," or "no sustained shift of health state." Most patients (85%; n = 688) showed "no sustained shift," while 10% (n = 84) showed "improved" health state and only 4% (n = 34) had "worsened" over the length of the study.

The comparison of baseline characteristics for patients with and without sustained favorable long-term outcome over the 2-year postbaseline period are shown in Table 3. In general, the univariate analyses showed that patients with sustained favorable long-term outcome started out better compared with those without sustained favorable long-term outcome. At baseline, they were significantly more likely to have fewer symptoms, higher level of functioning, better quality of life, satisfaction with life, fewer medication-emergent events, and lower healthcare resource utilization.

**Table 3:** Baseline Characteristics by Sustained Favorable Long-Term Outcome (N = 1635)

	SUSTAINED FAVORABLE LONG-TERM OUTCOME		
	Yes n = 157	No n = 1478	Univariate p value
**SOCIO-DEMOGRAPHICS**			

Age, mean (sd)	42.09 (10.61)	42.31 (11.00)	.8182
Male, n (%)	77 (49.0)	924 (62.5)	.0010
Race/ethnicity, n (%)			
Caucasian	86 (54.8)	800 (54.1)	.7398
African-American	61 (38.9)	538 (37.8)	
Other	10 (6.4)	120 (8.1)	
Single marital status, n (%)	142 (91.0)	1324 (90.2)	.7380
High school education or less, n (%)	43 (27.6)	508 (34.6)	.0774
Employed, n (%)	66 (42.0)	271 (18.4)	<.0001
Lack of health insurance, n (%)	20 (12.7)	78 (5.3)	.0002
Family history, n (%)	80 (55.2)	776 (56.7)	.7198
Supervised housing, n (%)	24 (15.3)	483 (33.2)	<.0001

**DISEASE-RELATED AND SYMPTOMS**			

Age at illness onset, mean (sd)	21.50 (9.33)	19.97 (8.70)	.0446
Depression, mean (sd)	2.01 (0.88)	2.41 (1.01)	<.0001
MADRS total score, mean (sd)	9.89 (8.79)	14.68 (10.26)	<.0001
PANSS positive (symptom^a^), mean (sd)	14.89 (5.10)	18.84 (6.08)	<.0001
PANSS negative (symptom^a^), mean (sd)	14.10 (5.00)	18.72 (6.18)	<.0001
PANSS hostility (symptom^a^), mean (sd)	8.52 (3.40)	10.99 (3.63)	<.0001
PANSS disorganized (symptom^a^), mean (sd)	11.36 (3.51)	13.92 (4.52)	<.0001
PANSS anxiety/depression (symptom^a^), mean (sd)	9.02 (2.93)	10.71 (3.41)	<.0001
Remission (PANSS), n (%)	66 (43.1)	293 (20.0)	<.0001
PANSS total score, mean (sd)	57.79 (15.54)	72.58 (18.22)	<.0001
PANSS Bell factor, mean (sd)	11.22 (4.32)	13.78 (5.01)	<.0001
Psychosis, mean (sd)	1.63 (0.84)	2.01 (1.05)	<.0001
Vitality, mean (sd)	3.55 (1.17)	3.19 (1.29)	.0008

**FUNCTIONING/BEHAVIORS**			

Daily activities, mean (sd)	3.95 (0.95)	3.30 (1.21)	<.0001
Leisure activities, mean (sd)	3.18 (0.97)	2.64 (1.19)	<.0001
Social activities, mean (sd)	2.96 (0.98)	2.62 (1.06)	.0001
Social relationships, mean (sd)	3.17 (1.10)	2.82 (1.23)	.0005
Arrested^c^, n (%)	4 (2.5)	79 (5.4)	.1268
Violence, n (%)			
No thought of injuring anyone	152 (96.8)	1372 (93.1)	.0358
Thoughts of injuring someone	0	60 (4.1)	
Have injured someone	5 (3.2)	42 (2.8)	
Victim of a crime, n (%)	8 (5.1)	166 (11.3)	.0170
Suicide thought/attempt, n (%)	18 (11.5)	249 (16.9)	.0789
Substance use, n (%)	44 (28.0)	386 (26.2)	.6262
Received helped with shopping, n (%)	59 (37.6)	840 (57.1)	<.0001
Received helped with leisure, n (%)	28 (17.8)	478 (32.5)	.0002
Received helped with household chores, n (%)	70 (44.6)	790 (53.6)	.0309
Received helped with paying bills, n (%)	77 (49.0)	899 (61.1)	.0035
Received helped with job search, n (%)	29 (18.5)	246 (16.8)	.5940
Received helped with getting benefits, n (%)	39 (25.2)	472 (32.1)	.0782
Received helped with legal issues, n (%)	11 (7.1)	142 (9.7)	.3046
General life satisfaction, mean (sd)	5.02 (1.47)	4.53 (1.59)	.0002
Satisfaction with basic needs, mean (sd)	5.23 (1.04)	4.71 (1.12)	<.0001
Satisfaction with social life, mean (sd)	5.08 (0.85)	4.57 (1.05)	<.0001
SF 12-Mental Health, mean (sd)	45.22 (12.44)	40.45 (13.34)	<.0001
SF 12-Physical Health, mean (sd)	47.70 (11.29)	45.17 (12.99)	.0192
Occupational role functioning (QLS 9), mean (sd)	3.15 (1.88)	1.76 (1.73)	<.0001
Level of accomplishment (QLS 10), mean (sd)	3.86 (1.61)	2.23 (1.77)	<.0001
Mean QLS total, mean (sd)	3.71 (1.06)	2.74 (1.01)	<.0001
Global Assessment of Functioning (GAF), mean (sd)	52.59 (13.74)	40.48 (12.09)	<.0001
Overall impression of general health (good/very good/excellent), n (%)	116 (73.9)	885 (60.2)	.0008
Productivity, n (%)	138 (87.9)	916 (62.1)	<.0001

**MEDICATION ADHERENCE**			

Medication possession ratio < .80, n (%)	21 (13.9)	140 (9.9)	.1274
Non-adherence, n (%)	6 (3.9)	110 (7.6)	.0909

**MEDICATION-EMERGENT EVENTS**			

AIMS total, mean (sd)	2.28 (3.16)	3.52 (4.20)	.0005
Simpson-Angus total^d^, mean (sd)	2.93 (3.41)	4.61 (4.20)	<.0001
Patient-reported clearer thoughts from medication, mean (sd)	3.74 (1.19)	3.22 (1.35)	<.0001
Medication effects, mean (sd)	1.83 (0.63)	2.07 (0.75)	.0001
Restlessness, mean (sd)	1.68 (1.13)	1.87 (1.17)	.0533
Tardive dyskinesia, n (%)	33 (22.1)	489 (34.3)	.0027

**CONCOMITANT MEDICATION**			

Mood stabilizers, n (%)	35 (22.7)	469 (32.3)	.0145
Antidepressants, n (%)	58 (37.7)	573 (39.5)	.6541
Antiparkinsonians, n (%)	64 (41.6)	672 (46.3)	.2571
Anti-anxiety medications, n (%)	12 (7.8)	171 (11.8)	.1376

**HEALTHCARE RESOURCE UTILIZATION**			

Emergency service use ^b ^(acute care service), n (%)	11 (7.0)	174 (11.8)	.0711
Psychiatric hospitalization (acute care service) (past 4 weeks), n (%)	7 (4.5)	134 (9.1)	.0494
Number of hospitalizations (6 months), mean (sd)	0.13 (0.36)	0.31 (0.66)	.0010
Total number of days hospitalized (6 months), mean (sd)	2.29 (13.84)	5.82 (21.58)	.0453
Psychiatric hospitalizations (1-year), n (%)	34 (21.8)	509 (34.8)	.0011
Crisis call	2 (1.3)	73 (5.0)	.0362
Case Management ^d^, n (%)	5 (50.0)	190 (68.6)	.2158
Individual therapy, n (%)	139 (89.1)	1378 (94.1)	.0145

Abbreviations: AIMS = Abnormal Involuntary Movement Scale; MADRS = Montgomery-Åsberg Depression Rating Scale; n = number of patients; PANSS = positive and negative syndrome scale; QLS = Quality of Life Scale; sd = standard deviation; SF = short form.

^a ^PANSS factors per Marder et al. (1997) [29]

^b ^Emergency use was both patient reported for the past 4 weeks and from the past 6 months recorded in the medical record.

^c ^The variation inflation factor for the arrested measure was greater than 10 and therefore not included in the stepwise logistic regression model.

^d ^Measure was not included in the stepwise logistic regression due to missing data.

NOTE: The univariate comparison of baseline characteristics for patients with and without sustained favorable long-term outcome over the 2-year postbaseline period.

**Figure 2:**
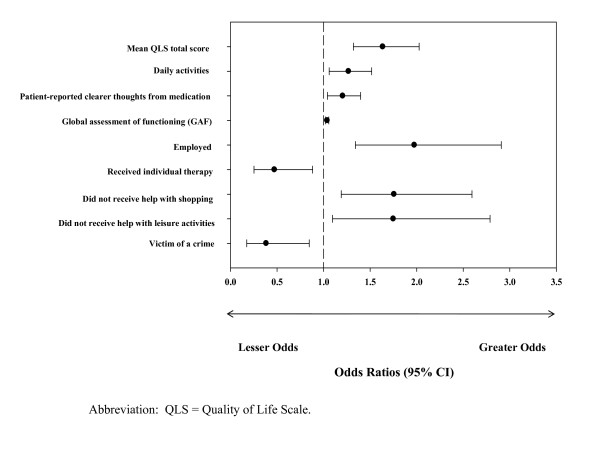
**The best baseline predictors of sustained favorable long-term outcome over a 3-year period**. When assessing the association between all baseline measures and sustained favorable long-term outcome, only 9 variables remained statistically significant.

When assessing the association (OR [95% CI]) between all baseline measures and sustained favorable long-term outcome in the last step of the analysis, only 9 variables remained statistically significant (Figure 2). Patients who were employed (1.98 [1.34, 2.91]), shopped without receiving assistance (1.76 [1.19, 2.59]), and engaged in leisure activities without receiving assistance (1.75 [1.10, 2.79]) had significantly greater odds of experiencing sustained favorable long-term outcome, while those who received individual therapy (0.47 [0.25, 0.88]) and were victims of a violent or non-violent crime (0.38 [0.17, 0.85])had significantly lower odds of experiencing sustained favorable long-term outcome. In addition, patients experiencing clearer thoughts from their medication (1.21 [1.04, 1.40]), a better quality of life (mean QLS total score: 1.64 [1.32, 2.03]), better global functioning (1.04 [1.02, 1.06]), and more daily activities (1.27 [1.06, 1.52]) had significantly greater odds of experiencing sustained favorable long-term outcome.

## Discussion

Using data from a large 3-year prospective observational study, this analysis identified 5 distinct health state clusters among chronically ill patients with schizophrenia treated in usual care settings in the US. This analysis incorporated its definition of patients' health state, severity of symptoms level of functioning, and use of acute care services, thus reflecting a broader health state concept that is not confined to symptomatology alone. Although the concept of broadening the definition of outcome has been utilized in a few prior schizophrenia studies, these studies have incorporated only patient's level of functioning along with symptoms [13-15]. To our knowledge, incorporating the patient's use of acute care services, severity of symptoms, and level of function has not been previously explored in the literature and provides a holistic view of the health status of the patient.

In this study, only 10% of the patients achieved "sustained favorable long-term outcome" over a 2-year period. A further assessment of cluster shift over the 3-year study period showed that a few patients (10%) improved over time (based on the definition of sustained favorable outcome), while the majority of patients (85%) had no sustained change from baseline in health state. Current findings suggest there continues to be a great need for improvement in the health status, and thus the need for better treatments, of these chronically ill patients with schizophrenia. This is a consistent message from past research, although this current study shows that a rather small percentage of patients are achieving "sustained long-term favorable outcome." Past research, which used a different definition of outcome and a different study duration, has have shown that current medications are effective for only approximately 50% of patients [4-6].

Our findings may reflect a somewhat conservative definition of "favorable and sustained long-term outcome," considering we required patients to be closest to the best baseline health status cluster in each of the 2 years following the initial cluster assignment baseline assessment. Therefore, there is the possibility that more than 10% of patients have improved, just not to the degree defined in this study as "sustained favorable long-term outcome."

Importantly, this study identified a small set of baseline characteristics that predict long-term sustained favorable health states over the study period. These characteristics included better quality of life, more daily activities, patient-reported clearer thinking from medication, better global functioning, being employed, not receiving individual therapy, not being a victim of a crime, and receiving less help with shopping or leisure activities. In general, when exploring baseline factors that were associated with sustained favorable long-term outcome, patients with a less severe illness profile (i.e., quality of life and functioning) were more likely to subsequently experience the favorable outcome of interest. Results of this study are consistent with prior research using data from patients in clinical trial settings, which found that patients with a less severe illness profile at baseline (e.g., higher level of functioning) had more favorable outcomes [14,31]. A naturalistic study [15] also confirmed that characteristics of those patients who were functioning better at baseline was predictive of functional remission.

Of particular interest, the results indicate that more daily activities and receiving less help with shopping or leisure activities were associated with sustained favorable long-term outcome. These factors are potentially modifiable and easy to assess, thus enabling clinicians to better understand and help optimize the treatment plan for the patient.

Although prior studies utilized varying endpoints, methods, and research designs, results appear similar to the present study in that patients who had better baseline profiles appeared to have better outcomes. The present study expands on past research by exploring potential predictors of sustained favorable long-term outcome and by using a broadly defined outcome measure that combines symptom severity, level of functioning, and use of acute care sevices. Moreoever, since data from randomized controlled trials provide efficacy data in a relatively homogenous population under artifical circumstances, it is reassuring to find that these results are confirmed in usual practice real-world settings. The identification of predictors of favorable outcomes suggests that clinicians could make early projections of health states and identify those patients more likely to achieve favorable long-term outcomes, enabling early therapeutic interventions to enhance benefits for patients.

This study has a number of limitations, including infrequent assessments. Clinician-reported outcomes were obtained only annually, and patient-reported outcomes were assessed every 6 months. Due to the infrequent assessments and the fact that schizophrenia is an illness characterized by relapses and remissions, this study was unable to capture episodic exacerbations or relapses that may have occured between assessments. Because of these infrequent assessments, other changes within those time periods and the potentially important variable of patients' early response to therapy were not captured. Lack of early response (week 2) has been found to be an important predictor [2,3] of subsequent response to therapy. There may be additional predictors not assessed, thus not explored, such as premorbid functioning, that may play an important role in patients' long-term outcomes. Also, p-values presented in the univariate analysis results were not adjusted for multiple comparisons. This study was intended for hypothesis generating as opposed to confirmation of a hypothesis, so the results would need to be replicated in a different study. Further, due to the statistical modeling of a large number of potential predictors and the method used for that analysis, we employed multiple imputation methods for missing data to reduce the number of observations deleted from the analysis. While the analysis included most (83%) of the enrolled patients, the patients who were included in the baseline cluster analysis were found to have a milder illness profile compared with those who were excluded, and the likely impact on the current results is unknown.

## Conclusions

This naturalistic, observational, 3-year study of chronically ill patients with schizophrenia identified 5 distinct health state clusters, which incorporate patients' baseline symptom severity, level of functioning, and use of acute care services. Using these health states, this study identified a small set of baseline characteristics that best predict sustained favorable long-term outcome over a 2-year postbaseline period. Findings may help clinicians tailor treatment alternatives that best meet individual patients' long-term needs. Understanding what factors may predict better long-term outcomes may also direct additional therapeutic options, enabling a holistic approach to treating patients and optimizing the potential benefit. Additional research building upon the current findings may allow for identification of early therapeutic interventions that could enhance patients' likelihood of achieving sustained favorable long-term outcomes.

## Competing interests

The authors, Drs. Cuyún Carter, Faries, and Ascher-Svanum and Ms. Milton, are full-time employees and minor stockholders of Eli Lilly and Company and/or its subsidiaries.

## Authors' contributions

GBCC, DRM, HA-S, and DEF contributed to the design of the study. DRM performed the statistical analyses. GBCC, DRM, HA-S, and DEF helped to draft the manuscript and approved the final version.

## Pre-publication history

The pre-publication history for this paper can be accessed here:

http://www.biomedcentral.com/1471-244X/11/143/prepub

## References

[B1] Stroup TS Heterogeneity of treatment effects in schizophrenia Am J Med 2007 120 Suppl 1 S26 S31 17403379 10.1016/j.amjmed.2007.02.005

[B2] Kinon BJ Chen L Ascher-Svanum H Stauffer VL Kollack-Walker S Sniadecki JL Kane JM Predicting response to atypical antipsychotics based on early response in the treatment of schizophrenia Schizophr Res 2008 102 230 240 18423985 10.1016/j.schres.2008.02.021

[B3] Ascher-Svanum H Nyhuis AW Faries DE Kinon BJ Baker RW Shekhar A Clinical, functional, and economic ramifications of early nonresponse to antipsychotics in the naturalistic treatment of schizophrenia Schizophr Bull 2008 34 1163 1171 10.1093/schbul/sbm134 18156640 PMC2632496

[B4] Kerwin RW Osborne S Antipsychotic drugs Medicine 2000 28 23 25

[B5] Lieberman JA Stroup TS McEvoy JP Swartz MS Rosenheck RA Perkins DO Keefe RS Davis SM Davis CE Lebowitz BD Severe J Hsiao JK Clinical Antipsychotic Trials of Intervention Effectiveness (CATIE) Investigators Effectiveness of antipsychotic drugs in patients with chronic schizophrenia N Engl J Med 2005 353 1209 1223 10.1056/NEJMoa051688 16172203

[B6] Miyamoto S Duncan GE Marx CE Lieberman JA Treatments for schizophrenia: a critical review of pharmacology and mechanisms of action of antipsychotic drugs Mol Psychiatry 2005 10 79 104 10.1038/sj.mp.4001556 15289815

[B7] Ayuso-Gutiérrez JL del Río Vega JM Factors influencing relapse in the long-term course of schizophrenia Schizophr Res 1997 28 199 206 10.1016/S0920-9964(97)00131-X 9468354

[B8] Perkins DO Predictors of noncompliance in patients with schizophrenia J Clin Psychiatry 2002 63 1121 1128 10.4088/JCP.v63n1206 12523871

[B9] Thieda P Beard S Richter A Kane J An economic review of compliance with medication therapy in the treatment of schizophrenia Psychiatr Serv 2003 54 508 516 10.1176/appi.ps.54.4.508 12663838

[B10] Liu-Seifert H Adams DH Kinon BJ Discontinuation of treatment of schizophrenic patients is driven by poor symptom response: a pooled post-hoc analysis of four atypical antipsychotic drugs BMC Med 2005 3 21 10.1186/1741-7015-3-21 16375765 PMC1327673

[B11] Levine SZ Leucht S Elaboration on the early-onset hypothesis of antipsychotic drug action: treatment response trajectories Biol Psychiatry 2010 68 86 92 10.1016/j.biopsych.2010.01.012 20227681

[B12] Rabinowitz J Levine SZ Haim R Häfner H The course of schizophrenia: progressive deterioration, amelioration or both? Schizophr Res 2007 91 254 258 10.1016/j.schres.2006.12.013 17293084

[B13] Lambert M Schimmelmann BG Naber D Schacht A Karow A Wagner T Czekalla J Prediction of remission as a combination of symptomatic and functional remission and adequate subjective well-being in 2960 patients with schizophrenia J Clin Psychiatry 2006 67 1690 1697 10.4088/JCP.v67n1104 17196047

[B14] Lipkovich IA Deberdt W Csernansky JG Buckley P Peuskens J Kollack-Walker S Rotelli M Houston JP Defining "good" and "poor" outcomes in patients with schizophrenia or schizoaffective disorder: a multidimensional data-driven approach Psychiatry Res 2009 170 161 167 10.1016/j.psychres.2008.09.004 19897252

[B15] Schennach-Wolff R Jäger M Seemüller F Obermeier M Messer T Laux G Pfeiffer H Naber D Schmidt LG Gaebel W Huff W Heuser I Maier W Lemke MR Rüther E Buchkremer G Gastpar M Möller HJ Riedel M Defining and predicting functional outcome in schizophrenia and schizophrenia spectrum disorders Schizophr Res 2009 113 210 217 10.1016/j.schres.2009.05.032 19560901

[B16] Ascher-Svanum H Faries DE Zhu B Ernst FR Swartz MS Swanson JW Medication adherence and long-term functional outcomes in the treatment of schizophrenia in usual care J Clin Psychiatry 2006 67 453 460 10.4088/JCP.v67n0317 16649833

[B17] Lehman AF Fischer EP Postrado L Delahanty J Johnstone BM Russo PA Crown WH The Schizophrenia Care and Assessment Program Health Questionnaire (SCAP-HQ): an instrument to assess outcomes of schizophrenia care Schizophr Bull 2003 29 247 256 14552500 10.1093/oxfordjournals.schbul.a007001

[B18] Lehman AF A quality of life interview for the chronically mentally ill Evaluation and Program Planning 1988 11 51 62 10.1016/0149-7189(88)90033-X

[B19] Fischer EP Mental health outcome module development and testing: development and initial validation of the schizophrenia outcomes module - final report, SDR Grant #91,005 1993 Washington, DC: Department of Veterans Affairs

[B20] Cuffel BJ Fischer EP Owen RR Jr Smith GR Jr An instrument for measurement of outcomes of care for schizophrenia. Issues in development and implementation Eval Health Prof 1997 20 96 108 10.1177/016327879702000107 10183315

[B21] Ware J Jr Kosinski M Keller SD A 12-Item Short-Form Health Survey: construction of scales and preliminary tests of reliability and validity Med Care 1996 34 220 233 10.1097/00005650-199603000-00003 8628042

[B22] Mayfield D McLeod G Hall P The CAGE questionnaire: validation of a new alcoholism screening instrument Am J Psychiatry 1974 131 1121 1123 4416585 10.1176/ajp.131.10.1121

[B23] Kay SR Fiszbein A Opler LA The positive and negative syndrome scale (PANSS) for schizophrenia Schizophr Bull 1987 13 261 276 3616518 10.1093/schbul/13.2.261

[B24] Montgomery SA Åsberg M A new depression scale designed to be sensitive to change Br J Psychiatry 1979 134 382 389 10.1192/bjp.134.4.382 444788

[B25] Simpson GM Angus JW A rating scale for extrapyramidal side effects Acta Psychiatr Scand Suppl 1970 212 11 19 4917967 10.1111/j.1600-0447.1970.tb02066.x

[B26] Guy W ECDEU Assessment manual for psychopharmacology, revised 1976 Rockville, MD: US Department of Health, Education, and Welfare Publication ADM 76-338

[B27] American Psychiatric Association Diagnostic and Statistical Manual of Mental Disorders 1994 Fourth Washington, DC: American Psychiatric Association

[B28] Heinrichs DW Hanlon TE Carpenter WT Jr The Quality of Life Scale: an instrument for rating the schizophrenic deficit syndrome Schizophr Bull 1984 10 388 398 6474101 10.1093/schbul/10.3.388

[B29] Marder SR Davis JM Chouinard G The effects of risperidone on the five dimensions of schizophrenia derived by factor analysis: combined results of the North American trials J Clin Psychiatry 1997 58 538 546 10.4088/JCP.v58n1205 9448657

[B30] Ward JH Hierarchical grouping to optimize an objective function J Amer Statistical Assoc 1963 58 236 244 10.2307/2282967

[B31] Levine SZ Rabinowitz J Trajectories and antecedents of treatment response over time in early-episode psychosis Schizophr Bull 2010 36 624 632 10.1093/schbul/sbn120 18849294 PMC2879688

